# Impact of L-arginine and L-citrulline supplementation on macrophage responses to *Mycobacterium tuberculosis*

**DOI:** 10.3389/fimmu.2026.1810985

**Published:** 2026-05-05

**Authors:** Gunapati Bhargavi, Sathyavageeswaran Shreeram, Guenolee Prioult, Ranjeet Kumar, Selvakumar Subbian

**Affiliations:** 1 Public Health Research Institute, New Jersey Medical School, Rutgers University, Newark, NJ, United States; 2 Nestle Health Science, Bridgewater, NJ, United States

**Keywords:** amino acids, antibiotics, antimicrobial response, gene expression, host directed therapy, infection, macrophages, tuberculosis

## Abstract

L-arginine (ARG) availability is a critical determinant of macrophage antimicrobial capacity, as it fuels nitric oxide production and other immune effector pathways essential for restricting *Mycobacterium tuberculosis* (Mtb), the causative agent of tuberculosis (TB). L-citrulline (CIT), a precursor in the ARG regeneration cycle, can replenish intracellular ARG pools when transport is limited. However, the comparative and combined effects of exogenous ARG and/or CIT on intracellular Mtb control across macrophage lineages and activation states remain insufficiently defined. This study investigated how supplementation with ARG, CIT or their combination influences Mtb survival in human and murine, primary macrophages and cell line, both in naïve and IFNγ-activated states, and evaluated whether these amino acids can enhance the activity of anti-TB drugs, isoniazid (INH) and rifampicin (RIF). Across a 5-day infection course, both ARG and CIT significantly reduced intracellular Mtb loads relative to untreated cells, with high-dose supplementation eliciting earlier and more sustained inhibition. These effects were amplified in IFNγ-stimulated macrophages, accelerating Mtb control and minimizing dose-dependent differences. Combination of ARG plus CIT at intermediate doses produced additive benefits, most notably in murine macrophages where single-agent effects were limited. Co-supplementation with ARG or CIT improved early antimicrobial effects of INH and RIF in all macrophage types, particularly under IFNγ stimulation. Gene expression analyses revealed coordinated metabolic and inflammatory reprogramming. For example, *TNF* expression was reduced by amino acid supplementation, while *IL6* expression was increased, and *NOS2* was significantly upregulated by ARG in IFNγ-stimulated cells, and *ARG1* expression was broadly suppressed in these cells. These findings demonstrate that ARG and CIT reshape macrophage antimicrobial response in a complementary manner, augmenting innate and drug-enhanced control of Mtb. The results support metabolic supplementation with ARG and CIT as a promising host-directed therapeutic approach to improve macrophage-mediated restriction of Mtb infection.

## 1 Introduction

*Mycobacterium tuberculosis* (Mtb) is a notorious bacterial pathogen responsible for an estimated 10.7 million new tuberculosis (TB) cases and approximately 1.23 million deaths globally in 2024 (1). The host immune response that is permissive or resistant to Mtb infection and progression to TB is not fully understood. The impact of nutritional supplements on TB has been a focus of extensive research over several decades; it has been established that balanced nutrition is vital for a healthy immune system to combat the progression of Mtb infection into active disease (2). Several studies have shown that disturbances in Vitamin A, C, D, and iron metabolism are associated with the reactivation of latent TB and an increased risk of disease (3, 4). In addition, micronutrients and amino acids have been shown to play potential roles in reducing the TB burden (5, 6).

Amino acids play critical roles during host cell-Mtb interactions at various stages of infection, as alterations in amino metabolism impact immune cell activation and host defense mechanisms (6, 7). Amino acids are essential nutrient sources for host immune cells during homeostasis and activation, as they contribute to metabolic pathways, supporting ATP production, and maintaining redox balance (8, 9). During Mtb infection, activation of the host immune cells, including macrophages and T cells, involves increased amino acid uptake and upregulation of amino acid transporters (10, 11).

Mtb primarily infects the alveolar macrophages in the lungs through complement receptors (CR1, CR3, and CR4), mannose receptors, and Toll-like receptors (TLRs), and signals macrophage activation (12–15). Infected macrophages form phagosomes around the bacterium, which fuse with the lysosome to form a phagolysosome complex in the cytoplasm (16). The phagolysosome typically destroys bacteria via acidic conditions by producing toxic molecules (17–19). One of the key antimicrobial responses elicited by Mtb-infected, activated macrophages is the elevated production of reactive nitric oxide (NO) intermediates, which contributes to bacterial control (20).

Although studies have reported the role of various amino acids in Mtb infection, L-Arginine (ARG) and L-Citrulline (CIT) have received more attention due to their involvement in NO-mediated anti-Mtb activities of macrophages (21). ARG is a semi-essential amino acid that serves as a precursor for NO synthesis, playing critical roles in cell division and immune function (22). Similarly, CIT is a non-protein-building amino acid structurally related to ARG, contributing to NO synthesis and ammonia detoxification. Upon Mtb infection, macrophages increase NO production through the enzyme inducible nitric oxide synthase (iNOS/NOS2), which converts ARG into NO and CIT (23). On the other hand, CIT, a byproduct of this reaction, is recycled to ARG via the arginosuccinate pathway, ensuring a continuous supply of ARG for NO synthesis (21). However, Mtb evades this defense by inhibiting NOS2 activity by depleting ARG availability, thereby weakening NO-mediated antimicrobial effects (21–24).

In the current study, we investigated the effect of ARG and/or CIT supplementation on the anti-Mtb response of human peripheral blood mononuclear cell-derived macrophages (hu-PBMCs), mouse bone marrow-derived macrophages (mo-BMDMs) and human THP-1 macrophage cell line with or without interferon-gamma (IFNγ) activation. We also tested the ability of ARG and CIT to augment the antibacterial effects of first-line anti-TB drugs, INH and RIF, in Mtb-infected macrophages. We observed distinct differences in intracellular Mtb survival between IFNγ-treated and untreated macrophages supplemented with various doses of ARG and CIT, either individually or in combination. A similar differential antimicrobial response was noted in these cells upon treatment with INH and RIF. Our findings suggest that ARG and CIT supplementation may promote the anti-Mtb effects of macrophages and have the potential to be a host-directed therapeutic (HDT) agent for TB.

## 2 Results

### 2.1 ARG and CIT differently affect the intracellular Mtb growth in various primary and THP-1 macrophages with or without IFN*γ* stimulation

**Figure 1:**
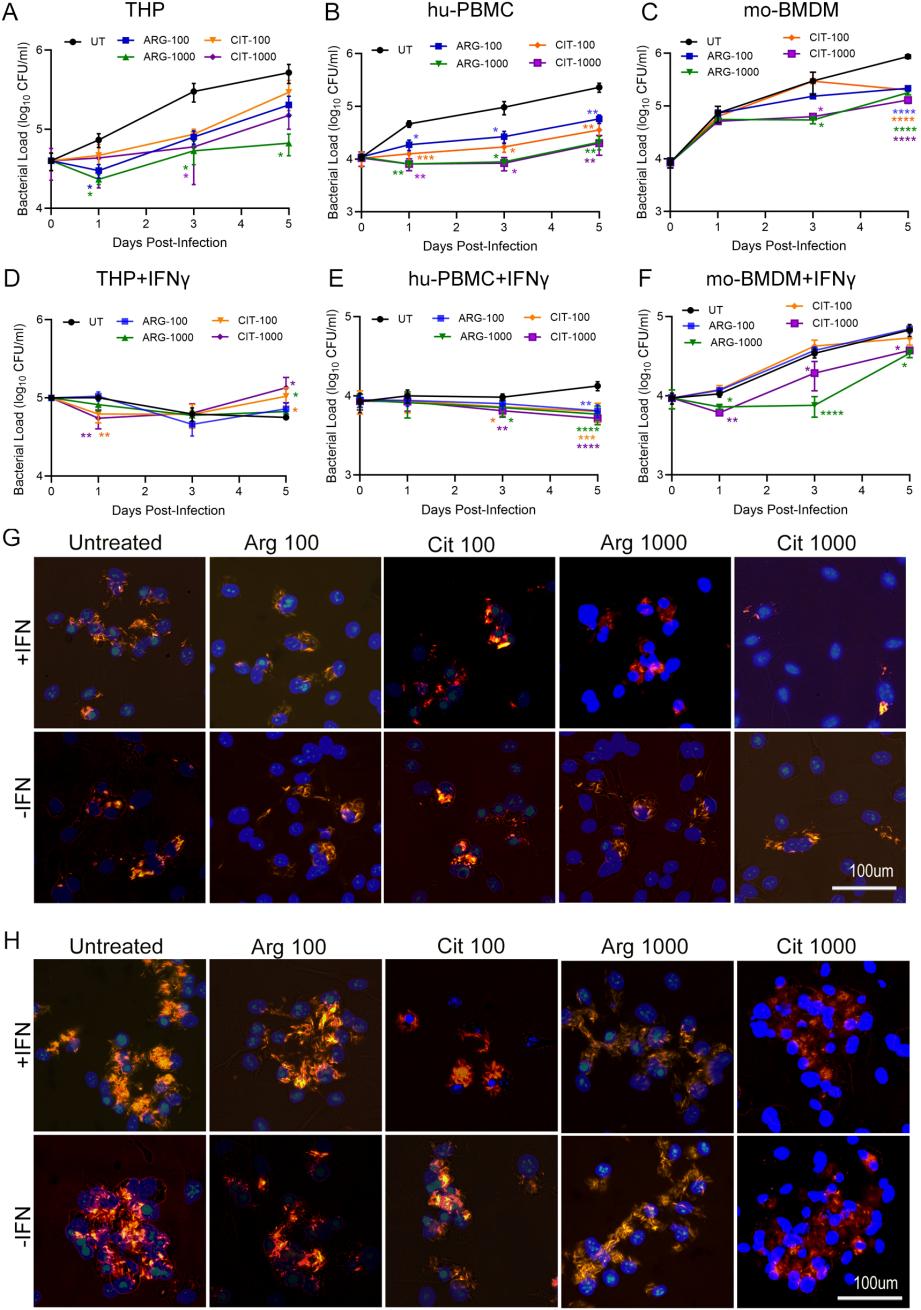
Effect of ARG and CIT on intracellular Mtb survival in macrophages stimulated with or without IFN*γ*. Unstimulated **(A–C)** or IFN*γ*-stimulated **(D–F)** macrophages were infected with Mtb and supplemented with 100μM or 1000μM of ARG or CIT, and Mtb load was determined up to 5 dpi. **(A, D)** Mtb load in THP-1 cells; **(B, E)** Mtb load in human peripheral blood-derived macrophages (hu-PBMC); **(C, F)** Mtb load in mouse bone marrow-derived macrophages (mo-BMDM). No-treatment (UT) was compared to individual treatment groups at each time point by Student’s T-test. Error bars indicate the standard error mean. *p<0.05, **p<0.01, ***p<0.005. **(G)** Representative microscopic imaging of IFN*γ*-stimulated (top row) or unstimulated (bottom row) THP-1 cells infected with Mtb (orange/red color) and supplemented with ARG or CIT at 3dpi. **(H)** Representative microscopic imaging of IFN*γ*-stimulated (top row) or unstimulated (bottom row) THP-1 cells infected with Mtb (orange/red color) and supplemented with ARG or CIT at 5dpi. Cells without any supplementation are included as control (Untreated). For **(G, H)**, 20–25 fields from triplicate wells (technical replicate), and were microscopically analyzed for Mtb, and the experiment was performed twice (biological replicate). Scale bar in **(G, H)** is 100 microns.

To determine the effect of exogenous ARG or CIT supplementation on Mtb growth control in macrophages, we quantified bacterial loads in hu-PBMCs, mo-BMDMs and THP cells following infection and compared four treatment groups (ARG-100 µM, ARG-1000 µM, CIT-100 µM, CIT-1000 µM) against untreated controls (UT) across a 5-day time course (**Figure 1**). The macrophages were either stimulated with IFNγ or not, prior to Mtb infection, and the doses of ARG and CIT were chosen based on previous studies that indicated rapid production of NO and associated control of Mtb load in macrophages at these doses (21, 25, 26).

As shown in **Figure 1**, both ARG and CIT supplementation significantly reduced intracellular Mtb load compared with untreated controls (UT) across all panels, with effects ranging from p < 0.05 to p < 0.0001, depending on dose, macrophage lineage, and time point post infection. In THP-1 cells without IFNγ stimulation, ARG-1000 µM and CIT-1000 µM showed a more consistent antibacterial effects, showing significant suppression at 1-, 3- and 5-days post infection (dpi) for ARG-1000 µM and 3 dpi for CIT-1000 µM (**Figure 1A**). Low-dose groups (ARG-100 µM and CIT-100 µM) produced sporadic early reductions but lost significance in late phases (**Figure 1A**). In hu-PBMCs, the rapid Mtb growth seen in UT was significantly reduced by ARG or CIT supplementation at all the tested doses, starting at 1 dpi and sustaining through 5 dpi. However, the low-dose groups exhibited a moderate effect, compared to the high-dose groups (**Figure 1B**). In mo-BMDMs, ARG-1000 µM and CIT-1000 µM significantly reduced bacterial load at 3 and 5 dpi, though low-dose groups did not predominantly reduce the Mtb load (**Figure 1C**). Together, these data establish that exogenous ARG or CIT can improve Mtb control relative to non-supplemented hu-PBMCs, mo-BMDM and THP-1 cells, with high-dose regimens delivering the largest effect sizes.

Since IFNγ stimulation leads to M1-type macrophage activation, we tested the hypothesis whether ARG and/or CIT would differentially affect Mtb growth in these activated macrophages. As expected, IFNγ stimulation altered the anti-Mtb response profile of macrophages, particularly THP-1 cells and hu-PBMCs across all tested doses of ARG and CIT. In IFNγ-stimulated THP-1 cells, significant reduction in Mtb load was noted on 1 dpi by CIT-100 µM and CIT-1000 µM supplementation, compared to UT; and all supplemented groups, except ARG-100 µM showed significant Mtb inhibition at 5 dpi (**Figure 1D**). Similarly, hu-PBMCs in all the supplementation groups showed significant reduction in Mtb load on 3 and 5dpi, relative to UT (**Figure 1E**). In contrast, mo-BMDMs exhibited a distinct pattern; while Mtb loads rose rapidly in UT conditions and remained high, supplementation with ARG-1000 µM or CIT-1000 µM significantly reduced the bacterial load at all the tested time points (**Figure 1F**). Compared to this, the low-dose supplementation yielded only a modest effect on Mtb load. Thus, IFNγ stimulation acts as a broad synergistic amplifier of macrophage control of Mtb growth, that advances onset, deepens magnitude, and equalizes performance across ARG and CIT doses, particularly in THP-1 and hu-PBMCs. Our findings also indicate the similarity between THP-1 cells and hu-PBMCs, which are consistent with, and justify the use of former cells as a surrogate of primary human cells in numerous Mtb studies (27–30). The effect of ARG and CIT supplementation in controlling Mtb load at the tested doses in THP-1 cells, with or without IFNγ stimulation, was also validated by qualitative microscopic analysis (**Figures 1G, H****).**

### 2.2 ARG or CIT supplementation enhances the effect of first line anti-TB drugs in macrophages with or without IFN*γ* stimulation

**Figure 2:**
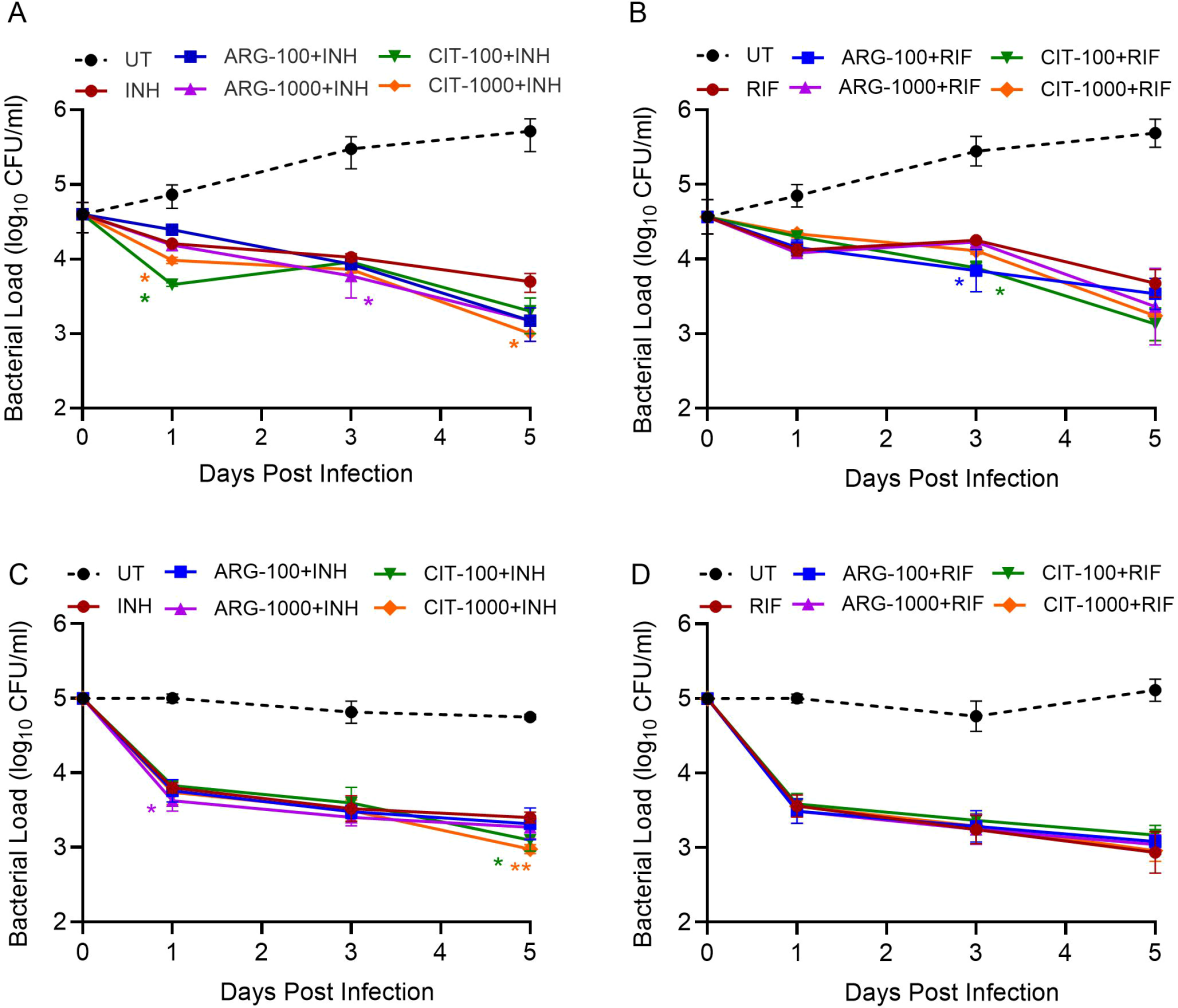
Impact of ARG and CIT combined with anti-TB drugs on intracellular Mtb survival in macrophages stimulated with or without IFN*γ*. Unstimulated **(A, B)** or IFN*γ*-stimulated **(C, D)** macrophages were infected with Mtb and supplemented with 100μM or 1000μM of ARG or CIT in-combination with INH **(A, C)** or RIF **(B, D)** and Mtb load was determined up to 5 dpi. In **(A–D)**, Mtb-infected macrophages without any treatment (untreated; UT), as well as treatment with antibiotics alone (INH or RIF) are included as controls. INH-only was compared to ARG+INH or CIT+INH **(A, C)**, while RIF-only was compared to ARG+RIF or CIT+RIF groups **(B, D)**, separately at each time point using Student’s T-test. Error bars indicate the standard error mean. *p<0.05, **p<0.01.

Next, we evaluated the effect of ARG in combination with INH and RIF, two of the most potent first-line anti-TB drugs, on Mtb survival in macrophages. We used the minimal inhibitory concentration (MIC) of INH and RIF for this assay. In naïve macrophages (no IFNγ stimulation), treatment with INH or RIF significantly reduced Mtb load compared to the no-antibiotics (UT) controls up to 5dpi (**Figures 2A, B**). In these macrophages, a significantly reduced Mtb load was noted at 1 dpi with CIT-100 µM+INH and CIT-1000µM+INH, while ARG-1000 µM+INH and CIT-1000 µM+INH significantly reduced the Mtb load on 3 and 5 dpi, respectively, compared to INH-only treated cells (**Figure 2A****).** Similarly, ARG-100 µM+RIF and CIT-100 µM+RIF treatment significantly reduced the Mtb load, compared to RIF-only treated cells (**Figure 2B**).

In IFN*γ*-stimulated macrophages, ARG-1000 µM+ INH significantly reduced Mtb load at 1dpi, while CIT-100 µM+INH and CIT-1000µM+INH supplement significantly reduced the Mtb survival at 5dpi, compared to INH-only treated macrophages (**Figure 2C**). In contrast, addition of RIF to ARG or CIT at the tested doses did not significantly affect the Mtb load, compared to RIF-only treated group (**Figure 2D**). Overall, the Mtb killing patterns reveal that (i) ARG or CIT supplementation with INH or RIF can confer early (1 and 3 dpi) benefit, particularly for IFN*γ*-stimulated macrophages; (ii) dose escalation enhances late effects for both amino acids (ARG-1000 µM and CIT-1000 µM with either INH or RIF); and (iii) CIT-1000 µM appears to be more consistently effective when combined with INH in reducing Mtb load at later stages of infection (i.e., 5 dpi).

### 2.3 Combination of ARG plus CIT supplementation has additive effect in controlling Mtb growth in macrophages stimulated with or without IFN*γ*

We tested the hypothesis that a combination of ARG plus CIT increase the macrophage Mtb killing than ARG or CIT alone. The rationale behind combining ARG and CIT, as ARG is a direct precursor of nitric oxide (NO), CIT serves as a precursor for ARG regeneration via the arginosuccinate pathway. Combining ARG and CIT was hypothesized to enhance and sustain NO production by ensuring a continuous supply of substrate, especially under conditions of increased metabolic demand during infection (31–33).

**Figure 3:**
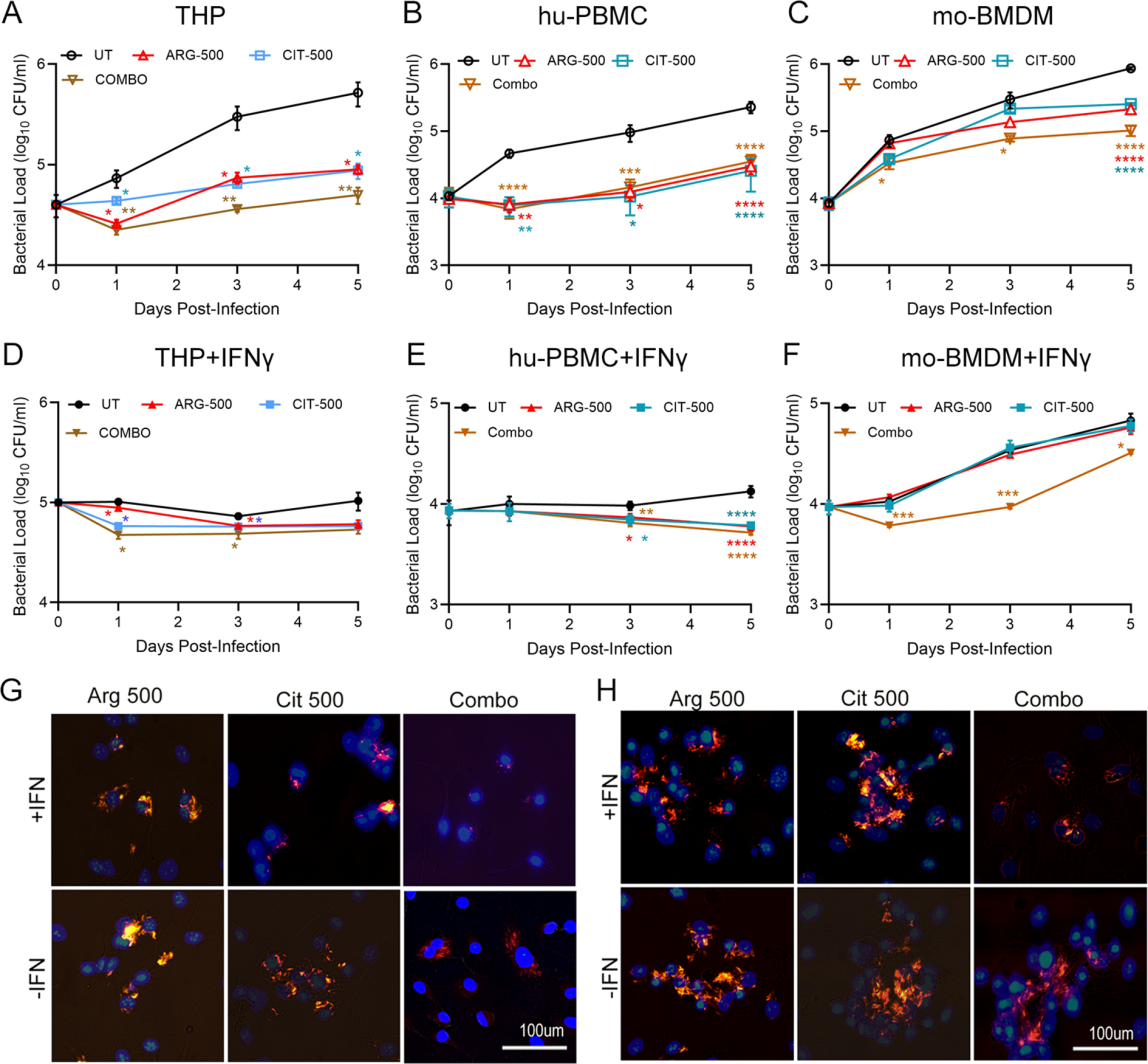
Effect of combined ARG and CIT supplementation on intracellular Mtb survival in macrophages stimulated with or without IFN*γ*. Unstimulated **(A–C)** or IFN*γ*-stimulated **(D–F)** macrophages were infected with Mtb and supplemented with 500μM each of ARG or CIT individually or in-combination (COMBO), and Mtb load was determined up to 5 dpi. **(A, D)** Mtb load in THP-1 cells; **(B, E)** Mtb load in hu-PBMC; **(C, F)** Mtb load in mo-BMDM. **(G)**. Representative microscopic imaging of IFN*γ* stimulated (top row) or unstimulated (bottom row) macrophages infected with Mtb (orange/red color) and supplemented with ARG or CIT, individually or in-combination at 3dpi. **(H)** Representative microscopic imaging of IFN*γ*-stimulated (top row) or unstimulated (bottom row) THP-1 cells infected with Mtb (orange/red color) and supplemented with ARG or CIT individually or in-combination at 5dpi. Mtb-infected macrophages without any supplementation are included as control (UT). For **(G, H)**, the experiment was performed twice in triplicate wells, and 20–25 fields were microscopically analyzed for Mtb. Scale bars in **(G, H)** are 100 microns. No-treatment group (UT) was compared to individual treatment groups at each time point using Student’s T-test. Error bars indicate the standard error mean. *p<0.05, **p<0.01, ***p<0.005, ****p<0.001.

We chose an intermediate dose (i.e, 500μM) of ARG and CIT between 100 and 1000 μM, and tested the effect of supplementation either individually, or in combination (COMBO; mix of 500μM each of ARG and CIT) (**Figure 3**) in hu-PBMCs, mo-BMDMs and THP-1 macrophages with or without prior IFNγ stimulation. This 500μM dosing would also be useful to test the additivity of ARG+CIT against supplementation with 1000 µM of individual amino acids (**Figures 1**, **2**). In the naïve (i.e., no IFNγ stimulation) hu-PBMCs and THP-1 macrophages, supplementation with 500μM ARG or CIT individually or in combination (COMBO), significantly reduced the Mtb load compared to the non-supplemented control (UT; untreated), at all the tested time points (**Figures 3A, B**). The COMBO supplementation group also significantly reduced the Mtb load in mo-BMDMs at all the tested time points. However, ARG-500 µM and CIT-500 µM groups showed significant Mtb control only on 5 dpi, compared to the non-supplemented (UT; untreated) group (**Figure 3C**).

In IFNγ-stimulated hu-PBMCs and THP-1 macrophages, but not in mo-BMDMs, the Mtb load reduced in general (**Figures 3D–F**). The Mtb load was significantly reduced in the ARG-500 µM, CIT-500 µM and COMBO groups at 1 and 3 dpi, compared to UT group (**Figure 3D**). A similar result was also noted in hu-PBMCs but on 3 and 5 dpi (**Figure 3E**). In contrast, only the COMBO group showed significant Mtb control in mo-BMDMs, compared to non-supplemented cells, at all the tested time points (**Figure 3F**). Using microscopic imaging, we further validated and confirmed the differential Mtb killing in THP-1 macrophages with or without prior IFNγ stimulation and supplementation with 500μM ARG or 500μM CIT or COMBO on 1 and 5dpi (**Figures 3G, H**). Overall, these observations suggest that supplementation with ARG+CIT improved the control of Mtb growth in IFNγ-stimulated and unstimulated macrophages, compared to individual amino acid, although the effect was more pronounced in IFNγ-stimulated cells.

### 2.4 Combination of ARG plus CIT augments the effect of first line anti-TB drugs on Mtb growth in macrophages with or without IFN*γ* stimulation

**Figure 4:**
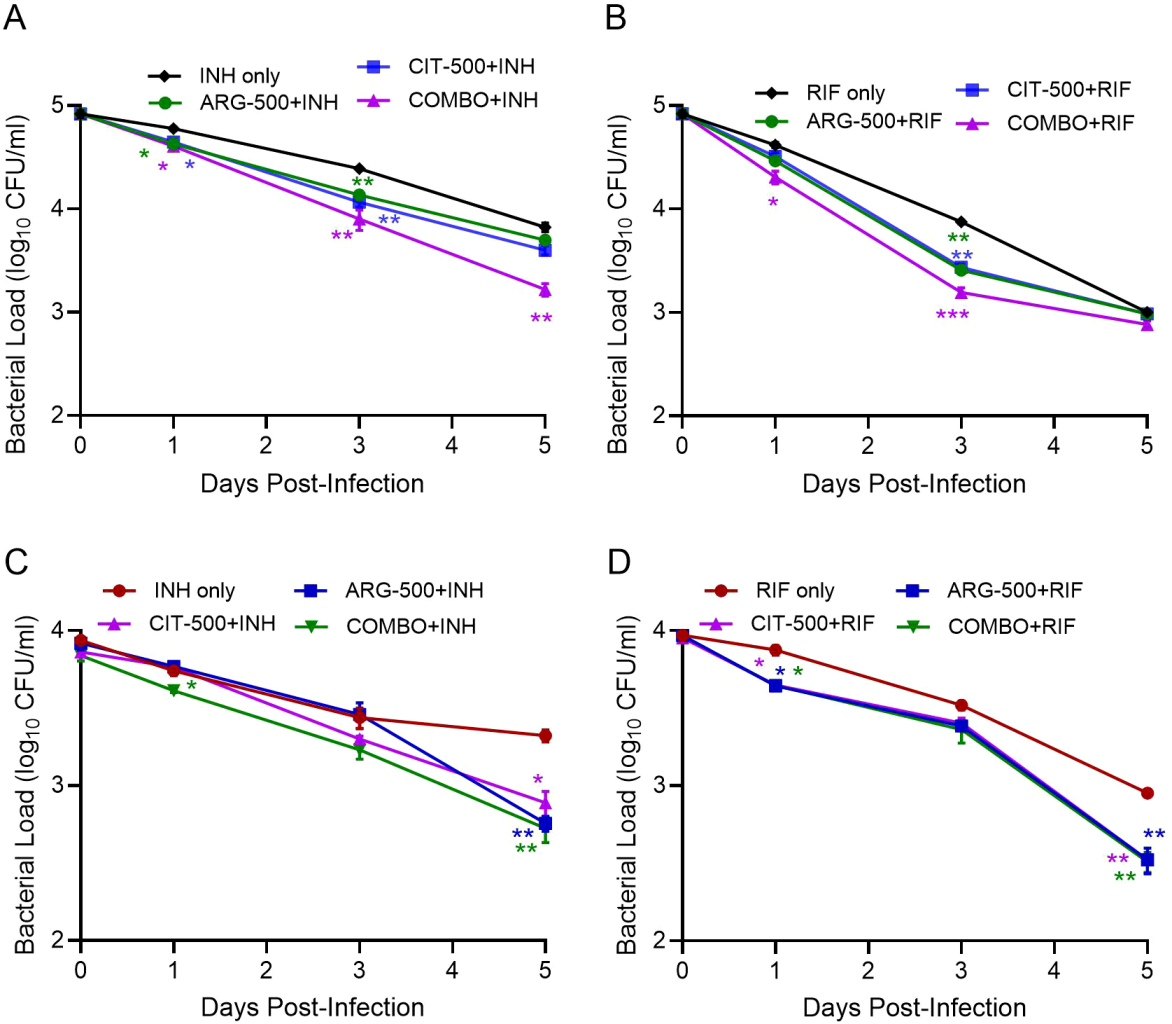
Impact of ARG plus CIT combined with anti-TB drugs on intracellular Mtb survival in macrophages stimulated with or without IFN*γ* Unstimulated **(A, B)** or IFN*γ*-stimulated **(C, D)** macrophages were infected with Mtb and supplemented with 500μM each of ARG or CIT individually or in-combination (COMBO) along with INH **(A, C)** or RIF (**B**, **D**), and Mtb load was determined up to 5dpi. Mtb-infected macrophages treated with INH only or RIF only were included as control. INH-only was compared to ARG+INH or CIT+INH or COMBO+INH groups **(A, C)** or RIF-only versus ARG+RIF or CIT+RIF or COMBO+RIF groups **(B, D)** at each time point using Student’s T-test. Error bars indicate the standard error mean. *p<0.05, **p<0.01, ***p<0.005.

We tested the Mtb killing effects of INH and RIF plus a combination of 500μM ARG and 500μM CIT (COMBO), on intracellular Mtb growth. In the naïve macrophages, COMBO+INH and ARG-500 µM+INH significantly reduced Mtb load on 1 and 3 dpi, compared to INH alone. However, the COMBO+INH showed significant reduction in Mtb load at all the tested time points. (**Figure 4A**). Similarly, the COMBO+RIF group significantly reduced the Mtb load at 1 and 3 dpi; whereas ARG-500 µM+RIF and CIT-500 µM+RIF showed significant reduction only at 3dpi, compared to RIF-only treated groups (**Figure 4B**). A similar trend in bacterial load reduction was noticed among various treatment groups in the IFNγ-stimulated macrophages infected with Mtb (**Figures 4C, D**). However, the magnitude of Mtb growth reduction was more pronounced in the IFNγ-stimulated, compared to unstimulated macrophages, particularly at 5 dpi (**Figures 4C, D**). In summary, supplementation with a combination of 500 µM each of ARG+CIT, with INH or RIF accelerates early and efficient Mtb control, compared to individual amino acid plus antibiotic supplementation, particularly in IFNγ-stimulated macrophages.

### 2.5 ARG and CIT differently affect the cytokine and antimicrobial gene expression in Mtb infected macrophages.

To determine the effect of ARG and CIT supplementation on the expression pattern of proinflammatory cytokines (TNF and IL6), antimicrobial marker (NOS2), and arginase-1 (ARG1) genes in Mtb-infected mo-BMDMs and THP-1 macrophages with or without prior IFNγ stimulation, we performed qPCR analysis at 5dpi (**Supplementary Figure 1**). We chose this time point based on the consistently reduced Mtb CFU in macrophages, as shown in **Figures 1**–**4**. *TNF, IL6, NOS2*, and *ARG1* were differentially expressed in naive and IFNγ-stimulated macrophages. In naive macrophages, supplementation with ARG or CIT significantly reduced *TNF* expression levels in a dose-dependent manner, compared to the non-supplemented, Mtb-infected cells. Furthermore, supplementation with 100 μM of ARG or CIT significantly reduced *TNF* expression, compared to 1000 μM of ARG or CIT (**Supplementary Figure 1A**). In IFNγ-stimulated macrophages, *TNF* expression was higher compared to unstimulated macrophages. However, ARG or CIT supplementation significantly reduced *TNF* expression levels irrespective of the dose, compared to non-supplemented cells, and there was no concentration-dependent reduction in *TNF* expression between ARG and CIT supplemented cells.

In contrast to *TNF*, the expression pattern of *IL6* was lower and similar between IFNγ-stimulated and unstimulated macrophages infected with Mtb. In both cell types, *IL6* expressions were significantly upregulated when supplemented with 100 or 1000 μM of ARG or 1000 μM of CIT, compared to no-supplementation (**Supplementary Figure 1A**). Similarly, *NOS2* expression was minimal in Mtb-macrophages without IFN*γ*-stimulation, irrespective of ARG or CIT supplementation. In contrast, Mtb-infected IFNγ-stimulated macrophages had a higher *NOS2* expression that further upregulated upon ARG supplementation, in a dose-dependent manner, reaching a statistically significant increase in ARG-1000 μM group. In contrast, CIT supplementation did not significantly affect NOS2 expression, compared to non-supplemented, Mtb-infected IFNγ-stimulated macrophages. The expression of *ARG1* was elevated in Mtb-infected naïve and IFNγ-stimulated macrophages. In Mtb-infected naïve macrophages, ARG-100 μM and CIT-1000 μM significantly reduced *ARG1* expression, while ARG-1000 μM or CIT-100 μM supplementation significantly increased *ARG1* expression, compared to non-supplemented infected cells. In contrast, *ARG1* expression was significantly reduced in Mtb-infected IFNγ-stimulated macrophages supplemented with ARG-100 μM or CIT-100 μM or CIT-1000 μM, compared to non-supplemented infected macrophages.

We further validated cytokine expression in Mtb-infected primary mo-BMDMs with or without IFNγ stimulation and with or without ARG or CIT supplementation. In unstimulated mo-BMDMs, ARG-100 and CIT-100 reduced the *TNF* expression, compared to 1000 μM of ARG or CIT, though the difference was not significant. In contrast, in IFN*γ*-stimulated, Mtb-infected mo-BMDMs, all the supplementation groups significantly reduced *TNF* expression compared to non-supplemented cells. However, *IL6* expression was higher in all supplemented groups, regardless of IFNγ stimulation, compared to non-supplemented group. In contrast, *NOS2* expression was minimal in Mtb infected macrophages both with and without IFNγ stimulation, though CIT-1000 μM supplementation increased *NOS2* expression compared to untreated controls in unstimulated macrophages. A dose-dependent increase in *NOS2* expression was also observed in unstimulated macrophages supplemented with 100 or 1000 μM of ARG or CIT. In IFNγ stimulated macrophages, *NOS2* expression remained comparable across all supplementation conditions, independent of concentration (**Supplementary Figure 1B**). *ARG1* expression was elevated in unstimulated macrophages supplemented with ARG-1000 μM or CIT-100 μM, compared with untreated controls. In contrast, in IFNγ stimulated macrophages, all ARG or CIT treated groups exhibited a statistically significant reduction in *ARG1* expression compared to non-supplemented, Mtb infected cells (**Supplementary Figure 1B**). Collectively, these data demonstrate that IFN*γ*-stimulation skews macrophages toward an M1-dominant transcriptional landscape, enhancing pro-inflammatory cytokine and *NOS2* expression.

## 3 Discussion

In this study, we evaluated the effects of ARG and/or CIT supplementation alone or in combination with or without anti-TB drugs, INH and RIF, on the macrophage viability to Mtb infection. Our data show that exogenous ARG and/or CIT supplementation improves macrophage control of intracellular Mtb across THP-1, hu-PBMC and mo-BMDM systems, with dose-dependent benefits that are amplified by IFNγ stimulation prior to infection. These findings are consistent with and supported by prior reports on canonical IFNγ biology, including the induction of NOS2/iNOS and nitric oxide (NO)–dependent antimicrobial programs, as well as the metabolic competition between iNOS and ARG1 for a shared ARG pool (21, 26, 32, 34). Mechanistically, IFNγ-primed macrophages rely on NOS2 to restrict Mtb *in vivo* and ex vivo, while NO constrains bacterial growth and Nos2−/− mice are highly susceptible to Mtb infection (34, 35). Consistent with this, we observed earlier and significant reductions in Mtb load in IFNγ-stimulated macrophages, particularly when ARG was provided at higher concentrations.

In IFNγ-activated settings, ARG supplementation directly increases the intracellular ARG pool available to upregulate NOS2, yielding more NO and efficient antimicrobial effects, which is consistent with our observation that ARG-1000 µM often outperforms lower doses and accelerates control (21, 23, 26, 34)). CIT contributes by recycling to ARG via arginosuccinate synthase 1 (ASS1) and arginosuccinate lyase (ASL) pathway; these enzymes catalyzes the conversion of CIT to ARG in a cell-autonomous manner and is crucial particularly when circulating ARG is in demand (21, 26, 31, 36). However, multiple studies indicate that recycling can be rate-limited and insufficient to sustain maximal NO, especially under strong inflammatory drive; this provides a plausible explanation for the weaker NOS2 upregulation we saw with CIT supplementation despite comparable Mtb loads in macrophages (21, 26, 31, 36). Indeed, classic macrophage work in J774 cells showed that CIT supplementation only partially restores NO in ARG-depleted conditions, whereas ARG robustly rescues NO production (31). Our findings extend these insights to human and murine primary macrophage systems and reinforce that substrate availability and cell state are complementary levers for antimycobacterial control.

Our studies also indicate IFNγ as a synergistic amplifier that transforms the macrophage response by boosting NOS2 and dampening ARG1, thereby favoring M1-like metabolism, which is associated with proinflammatory immune response needed for effective pathogen control, through the production of antimicrobial molecules such as NO (34, 35). In our study, the equalized dose performance and advanced onset of CFU reduction across different macrophage types. Prior studies on murine models of TB have established that NOS2 is essential for tuberculostasis and that NO tempers pathologic neutrophilic inflammation, thereby improving host control (34, 35). At the cellular level, IFNγ-activated macrophages can kill Mtb via NO-dependent mechanisms, including induction of apoptosis in infected cells, which is consistent with the stronger late-phase killing we observed after IFNγ plus amino acid supplementation (37). However, the anti-Mtb effects of ARG supplemented macrophages can occur through other pathways independent of NO (25, 38). Similarly, our study was focused on IFNγ-activated macrophages; however, the effect of ARG and/or CIT on IL-4-induced alternatively (M2)-activated macrophages is warranted to understand the complete mechanistic role of these amino acids in regulating antimicrobial responses in infected macrophages.

We noticed that across different macrophage types, the COMBO (500 μM each of ARG+ CIT) supplementation produced additive or superior Mtb control relative to single agents at the same dose, most notably in mo-BMDMs, where single 500 μM doses were weaker. A parsimonious explanation is complementary flux support, in which the ARG supplies immediate NOS2 substrate, while CIT buffers ARG availability through recycling when transporter capacity or catabolism (e.g., by ARG1) would otherwise limit NO synthesis (31–33). This model is also consistent with our observation that ARG1 expression dampens in IFNγ-stimulated macrophages with supplementation. Reduced ARG1 would remove a key sink for ARG pool and reinforce the M1 program in macrophages by inducing NOS2 expression as we observed here.

Although the response of various macrophage types to ARG and/or CIT supplementation were directionally consistent, the magnitude and timing varied by lineage in our study. While THP-1 and hu-PBMC macrophages behaved similarly in most settings, supporting the utility of THP-1 as a surrogate for primary human macrophages in intracellular Mtb assays (39, 40). In contrast, mo-BMDMs were more dose-dependent and benefited disproportionately from COMBO supplementation. This observation is consistent with the reported lineage differences in ASS1/ASL levels and activity, as well as ARG transport, and ARG1 activity (26, 32, 38).

Our transcriptional data showed reduced *TNF* expression with ARG or CIT in a dose-dependent manner in naïve cells and dose-independent fashion under IFNγ stimulation, which fits with prior observations that NO modulates TNF/IL-1β production in alveolar macrophages from TB patients (41). In contrast, *IL6* expression was upregulated with supplementation irrespective of IFNγ stimulation. It should be noted that one of Mtb’s virulence factor, ESAT-6, can drive macrophage IL-6 production via STAT3 pathway, and IL-6 signaling has complex, context-dependent effects on TB pathogenesis (e.g., host response modulation and IFNγ responsiveness) (32, 42). Furthermore, cytokine expression profiling in mouse BMDMs revealed that expression patterns of *TNF*, *IL6*, *NOS2*, and *ARG1* were comparable to those observed in the THP-1 infection model, supporting the consistency of host responses (43). These observations offer a potential route by which amino acid–dependent metabolic flux cross talks with inflammatory circuits that contribute to Mtb control in macrophages. Together, the expression pattern of cytokine genes observed in this study suggest that metabolic augmentation can enhance bactericidal pathways while avoiding hyperinflammation, particularly in IFNγ-primed macrophages where *ARG1* expression is kept in check and *NOS2* expression predominates.

We found that ARG and CIT accelerate and augment the killing effects of first-line anti-TB drugs (INH, RIF) in naïve and IFNγ-stimulated macrophages. These patterns fit the broader concept of host-directed therapy (HDT), in which metabolic or immunologic augmentation can sensitize intracellular bacilli to antibiotics-mediated killing (12, 44). Previous studies suggested the potential of ARG as adjunctive HDT for TB therapy (45). Along this line, we have previously shown that ARG supplementation upregulated NOS2 expression and mitophagy, and reduced lung damage in a murine model TB (46). Moreover, NO can shape the inflammatory milieu and limit neutrophil-dominant pathology, potentially improving local drug access over time (35). Taken together, our data justify further studies on dose–response matrices for INH/RIF + ARG and/or CIT to evaluate the synergy across various activation states of macrophages.

Furthermore, our results suggest that ARG and CIT supplementation, especially when combined, can aid in better control of intracellular Mtb in macrophages stimulated by IFNγ. In addition, these amino acids have the potential to be a feasible HDT adjunct to first-line chemotherapy for TB. The rationale for this claim is consistent with the findings that IFNγ activates an M1 program in macrophages (i.e, upregulation of NOS2 and downregulation of ARG1), while ARG fuels NO-dependent killing, and CIT stabilizes ARG availability via recycling, and the two together can improve bacterial control while modulating cytokines toward balancing the infection-induced inflammation (32, 34, 35, 41).

Our study has some limitations, such as the findings from *in vitro* systems cannot fully recapitulate granulomatous architecture or tissue pharmacology in an intact *in vivo* system. Similarly, while THP-1 mirrored hu-PBMC in most conditions, donor-to-donor variability in primary human macrophages, as well as differences between hu-PBMCs and mo-BMDMs warrants mixed-effects analyses in future work.

## 4 Materials and methods

### 4.1 Bacteria growth conditions

The original pathogenic *Mycobacterium tuberculosis* H37Rv (Mtb) strain was procured from American Type Culture Collection (ATCC, Manassas, VA, USA) and grown to mid-log phase (OD_600_ = 0.6-0.8) in Middlebrook 7H9 media supplemented with 10% albumin, dextrose, and catalase (ADC) (Difco, MI, USA), and stored at -80 ° C until ready to use. The number of bacterial colony-forming units (CFU) on the inoculum was enumerated by serially diluting the culture in 1x sterile PBS containing 0.05% Tween-80 and plating onto 7H10 Middlebrook agar (Hardy Diagnostics, CA, USA) supplemented with 10% ADC. The plates were incubated at 37 °C for 4–6 weeks and the number of bacterial CFUs was counted. For *in vitro* infection studies, stock vials were thawed and diluted in sterile 1x PBS and added to wells containing monocytes or macrophages. L-Arg, L-Cit, L-Lys as well as all other chemicals and antibiotics were purchased from Millipore-Sigma (Millipore-Sigma, CA, USA).

### 4.2 Macrophage growth conditions

Human blood monocytes were isolated from the whole blood of healthy volunteers (n=3) using Histopaq-1077 gradient centrifugation method and differentiated into macrophages (hu-PBMCs) by growing the monocytes in recombinant human M-CSF (50ng/ml; ThermoScientific, CA, USA) containing DMEM media supplemented with 10% fetal bovine serum (FBS; Phoenix Scientific, CA, USA). as we reported previously (30).

For mouse BMDMs, bone marrow monocytes from the tibia and femur of C57BL/6 mice (n=4) were isolated and grown in DMEM media supplemented with 10% FBS and recombinant mouse M-CSF (50ng/ml; ThermoScientific, CA, USA) as we reported previously (47). All animal procedures were approved by the Rutgers Institutional Animal Care and Use Committee (IACUC).

The human monocytic cell line THP-1 was originally obtained from ATCC and propagated in DMEM medium (ThermoScientific, CA, USA) supplemented with 10% FBS. The THP-1 cells were incubated with 10 nM phorbol 12-myristate 13-acetate (PMA; Millipore-Sigma, MO, USA) for 24 hours to differentiate into macrophages as we described previously (30).

For all the experiments in the current study, all macrophages (i.e, hu-PBMC, mo-BMDM and THP-1) were grown in DMEM for SILAC media (ThermoScientific, USA), which lacks L-Arg, L-Cit and L-Lys, and supplemented with 10% FBS.

### 4.3 ARG, CIT and anti-TB drugs

ARG and/or CIT were added to the cell growth media at 100, 500 or 1000 μM concentrations based on previous literature (21, 25, 26). In humans, fasting plasma concentrations of ARG and CIT are about 80–150 µM and 20–40 µM, respectively. Thus, 100 µM doses of ARG and CIT tested here are within physiological levels that are nutritionally achievable in humans (48). Although we used a media lacking ARG and CIT, the standard cell culture medium has about 400 µM (DMEM) to 1150 µM (RPMI) of ARG and no CIT. In humans, 1000 µM (1 mM) ARG, and not CIT, is supra-physiological plasma levels that cannot be achieved after taking oral supplementation (49). We tested these doses to determine whether there is any dose-dependent anti-Mtb effect in supplemented macrophages, in the context of HDT. Additionally, 1 ml of L-Lysine hydrochloride was separately added to the DMEM for SILAC media, as recommended by the manufacturer, and the complete media was sterilized using a 0.22-micron filter before use.

### 4.4 Intracellular Mtb survival assay

The intracellular survival assay to determine Mtb load was performed similarly to the adherence and uptake assays, except the infected cells were harvested at various time points post-infection (i.e., 1–5 dpi). Differentiated macrophages were seeded at 0.5x10^6^ cells/well in 24 well plates and were supplemented with 100, 500, or 1000 μM of ARG or CIT before infection. Cells were co-incubated with Mtb at an MOI of 3 for 3h. After infection, cells were washed with PBS and further incubated at 37 °C in 5% CO_2_ or lysed with 0.05% Triton X-100 in sterile 1x PBS (for T = 0). Ten-fold serial dilutions of the lysates were plated onto 7H10 OADC agar plates. These plates were incubated for 4 to 6 weeks, and CFUs were enumerated. The CFUs/ml were calculated and plotted as graphs.

### 4.5 Infection of Macrophages treated with ARG and CIT with or without anti-TB drugs

In a 24-well cell culture plate, macrophages were seeded at 0.5x10^6^ cells/well and treated with PMA for 24h. Following treatment, the cells were exposed to ARG and/or CIT in DMEM for SILAC media: ARG and CIT were added at 100, 500 or 1000 μM or 500 μM ARG + 500 μM CIT. The macrophages were subsequently infected with Mtb at an MOI of 3 for 3h, followed by washing of infected cells to remove extracellular Mtb, and the cells were replenished with SILAC media supplemented with the amino acids as above. To determine the effect of INH and RIF on ARG and/or CIT supplemented macrophages during Mtb infection, INH and RIF were added at their MIC of 0.1 µg/ml of INH and 0.1 µg/ml of RIF at 1 dpi. The bacterial load was assessed at 0, 1, 2, 3, and 5 dpi as mentioned above.

### 4.6 Intracellular Mtb Survival assay in IFN*γ*-stimulated macrophages with or without ARG and CIT supplementation

Macrophages were seeded at 0.25x10^6^ cells/well and activated with PMA for 24h in complete DMEM for SILAC media at 37° C in 5% CO_2._ The cells were stimulated with IFN*γ* 2ng/ml for 48h as reported earlier (25). The cells were supplemented with 100, 500, or 1000 μM of ARG or CIT, or a combination of 500 μM ARG + 500 μM CIT and infected with Mtb at an MOI of 3 for 3h. To determine the effect of INH and RIF on IFN*γ*-stimulated macrophages with or without ARG and CIT supplementation during Mtb infection, 0.1 µg/ml of INH and 0.1 µg/ml of RIF were added to wells at 24h post-infection. The bacterial load was assessed at 0, 1, 2, 3, and 5 dpi as mentioned above.

### 4.7 Microscopic analysis of Mtb in unstimulated or IFN*γ*-stimulated infected macrophages with ARG and CIT supplementation

Mtb within infected macrophages was detected using an Auramine–Rhodamine staining kit (Hardy Diagnostics, Santa Maria, CA, USA) following the manufacturer’s protocol. Briefly, macrophages were kept unstimulated or stimulated with IFNγ and seeded onto sterile glass coverslips placed in 24-well culture plates, treated with ARG or CIT as described above, and subsequently infected with Mtb at an MOI of 3 for 3 h. Uninfected and untreated macrophages served as negative controls. Following infection, both infected and uninfected macrophages were fixed overnight in 10% formalin, washed five times with sterile 1× PBS, and incubated in 70% ethanol overnight. Cells were then stained with Auramine–Rhodamine solution for 15 min, rinsed thoroughly with distilled water, and decolorized with 0.5% acid-alcohol for 3 min. After additional rinsing with water, the cells were counterstained with 1% potassium permanganate for 2 min. Stained coverslips were carefully removed, air-dried, mounted onto glass slides, and examined using a fluorescence microscope equipped with a 63× objective and images were captured as we described previously (30).

### 4.8 Cytokine gene expression analysis in infected macrophages

The crude lysates from uninfected and Mtb-infected macrophages were treated with 0.7 volume of Trizol, vortexed for 30 sec, and centrifuged at 10,000 rpm for 10 min. The supernatant was used for total host RNA extraction using the Qiagen Miniprep columns (Qiagen, CA, USA). Total RNA purified from infected and uninfected macrophages was used as a template for cDNA synthesis using the High-Capacity cDNA Reverse Transcription Kit following the manufacturer’s instructions (Applied Biosystems, Thermoscientific). The qRT-PCR assay was performed using 2X Universal SYBR Green Fast qPCR premix reagent (AB Clonal, USA) in an Agilent/AriaMx qPCR system (Agilent Technologies, USA) using primer pairs specific to *TNF*, *IL6*, *NOS2*, *ARG1* and *ACTNB*, which was used as internal control to normalize the expression values of target genes. The threshold cycle (Ct) values for individual cytokines were calculated using Agilent/Arial 1.71 software, and using the Ct values, we calculated the 2^ΔCt^, where ΔCt refers to a change in Ct between the targeted cytokine and internal control genes (48).

### 4.9 Statistical analysis

All the experiments were performed in three technical replicates (3 wells) and repeated at least twice (biological replicates). The hu-PBMCs were isolated from three healthy individuals (biological replicates) and used independently in two technical replicates. The mo-PBMCs isolated from two animals were pooled and used in triplicate wells (technical replicates) and the experiment was repeated twice (biological replicates). The Statistical data analysis was performed using GraphPad Prism 10 (GraphPad Prism, USA). Pairwise comparative analysis between control and single test groups was performed using Students’ t-test, and for multiple comparison analysis, one-way-ANOVA with Tukey’s correction method was used. For all the experiments, a p-value ≤ 0.05 was considered statistically significant.

## Data Availability

The raw data supporting the conclusions of this article will be made available by the authors, without undue reservation.

## Supplementary material

Supplementary Figure 1 Cytokine and antimicrobial gene expression in Mtb-infected macrophages stimulated with or without IFN*γ* and supplemented with ARG or CIT QPCR analysis of host genes coding for proinflammatory cytokines (*TNF*, *IL6*), antimicrobial (*NOS2*) and immune modulatory molecules (*NOS2*, *ARG1*) in Mtb-infected THP-1 **(A)** and mo-BMDMs **(B)** with or without ARG or CIT supplementation. Non-supplemented cells (UT) were included as control. Beta-actin gene transcripts (*ACTNB*) were used as housekeeping internal control. Fold changes were calculated and presented as relative to the levels of respective transcripts in uninfected macrophages. Values plotted are mean +/- SEM from two experiments done with triplicate samples. One-way ANOVA with Tukey’s correction was used for statistical analysis between untreated (UT) and different treatment groups. *p<0.05, **p<0.01, ***p<0.005.
