# Supplementary figures and images for: Impact of L-arginine and L-citrulline supplementation on macrophage responses to Mycobacterium tuberculosis
Source: Front Immunol. 2026 May 5;17:1810985. doi: 10.3389/fimmu.2026.1810985 (PMC13183539; doi:10.3389/fimmu.2026.1810985)

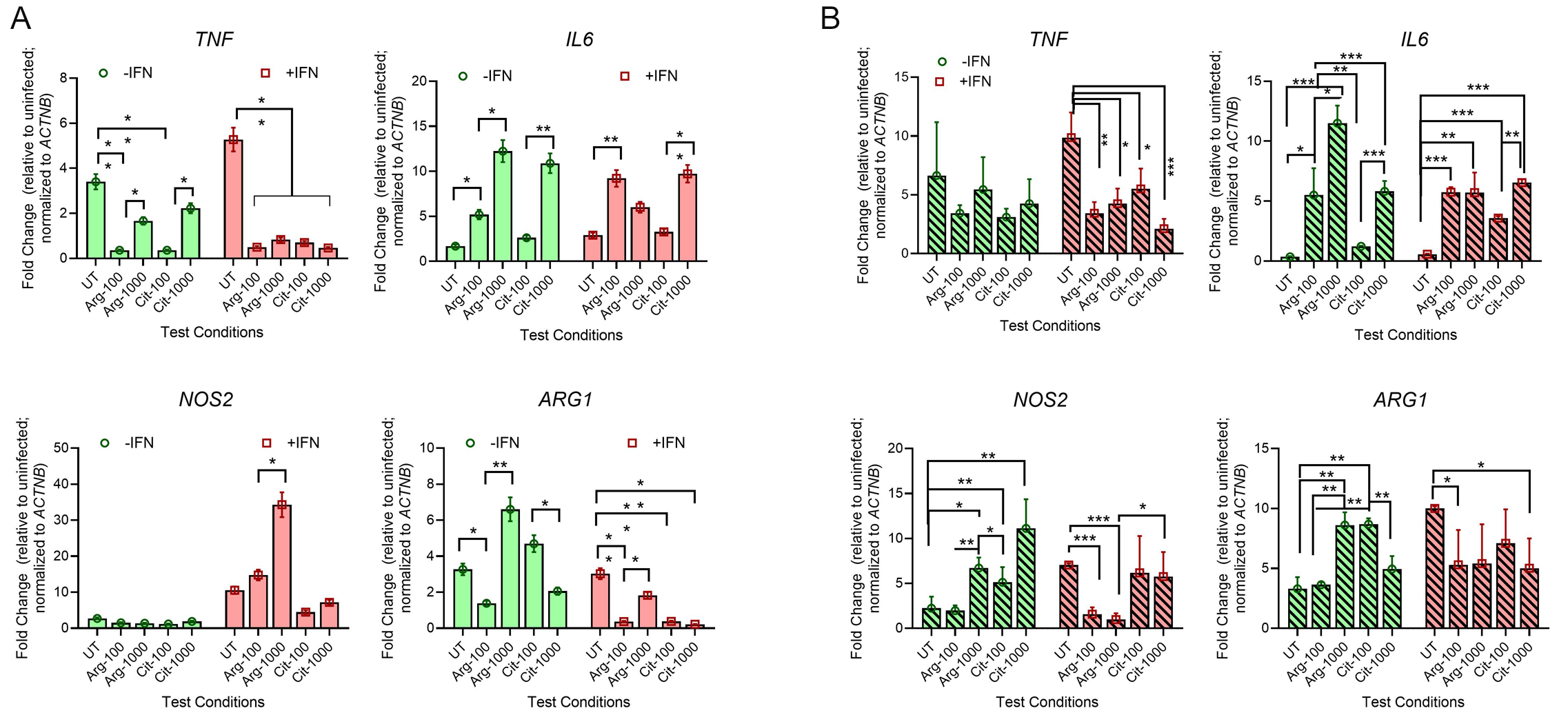

Supplement: Supplementary Figure 1 — Cytokine and antimicrobial gene expression in Mtb-infected macrophages stimulated with or without IFNγ and supplemented with ARG or CIT QPCR analysis of host genes coding for proinflammatory cytokines (TNF, IL6), antimicrobial (NOS2) and immune modulatory molecules (NOS2, ARG1) in Mtb-infected THP-1 (A) and mo-BMDMs (B) with or without ARG or CIT supplementation. Non-supplemented cells (UT) were included as control. Beta-actin gene transcripts (ACTNB) were used as housekeeping internal control. Fold changes were calculated and presented as relative to the levels of respective transcripts in uninfected macrophages. Values plotted are mean +/- SEM from two experiments done with triplicate samples. One-way ANOVA with Tukey’s correction was used for statistical analysis between untreated (UT) and different treatment groups. *p<0.05, **p<0.01, ***p<0.005. [file Image1.jpeg]
